# The Role of E2F Transcription Factor 1 Protein in Multiple Sclerosis (MS): A Comparative Study of Serum Levels in MS Patients and Healthy Controls

**DOI:** 10.7759/cureus.76889

**Published:** 2025-01-04

**Authors:** Emine Kılıçparlar Cengiz, Yasemin Ekmekyapar Fırat, Elif Onur, Tuba Denkçeken

**Affiliations:** 1 Neurology, Dr. Ersin Arslan Education and Research Hospital, Gaziantep, TUR; 2 Neurology, SANKO University School of Medicine, Gaziantep, TUR; 3 Medical Biology, SANKO University School of Medicine, Gaziantep, TUR; 4 Medical Biophysics, SANKO University School of Medicine, Gaziantep, TUR

**Keywords:** e2f1 levels, multiple sclerosis immunopathogenesis, multipl sclerosis, relapsing-remitting multiple sclerosis, secondary progressive multiple sclerosis

## Abstract

Background: This study investigates the role of E2F1 protein levels in patients with multiple sclerosis (MS). Genetic and environmental factors play a role in the pathogenesis of MS, with T and B lymphocytes contributing to tissue damage via immune responses. E2F1, a transcription factor, regulates the cell cycle in T cells and has been associated with autoimmune responses.

Methods: This cross-sectional study included 24 MS patients and 21 healthy controls matched for age and gender. Serum E2F1 levels were measured using enzyme-linked immunosorbent assay (ELISA). Clinical and imaging data were collected, including Expanded Disability Status Scale (EDSS) scores, MS subtype classification (RRMS, SPMS, PPMS), and lesion localization on MRI. Statistical analyses were performed to compare E2F1 levels across groups and explore associations with clinical parameters.

Results: It was observed that E2F1 levels were significantly elevated in the MS group compared to controls (p = 0.011). A subgroup analysis revealed higher E2F1 levels in relapsing-remitting MS (RRMS) than in progressive forms (SPMS and PPMS) (p = 0.024). In addition, female MS patients had significantly higher E2F1 levels than male MS patients (p = 0.028). No significant correlation was found between E2F1 levels and clinical variables such as disease duration or the EDSS score.

Conclusion: These findings indicate that E2F1 may play a role in MS pathogenesis, especially in the RRMS subtype, and may act as a biomarker for assessing inflammation and relapse susceptibility. Further research is needed to clarify the role of E2F1 in MS and its potential as a therapeutic target.

## Introduction

Multiple sclerosis (MS) is a chronic, inflammatory, demyelinating disease of the central nervous system. There is an interaction between genetic and environmental factors in the pathophysiology of MS [1]. It is known that T and B lymphocytes and microglia play a role in immunopathogenesis. T cells react excessively with autoantigens that damage central nervous system tissue. B cells can produce autoantibodies against autoantigens. In brain tissue, these cells can activate microglia and macrophages and thus promote local inflammation. It is assumed that the migration of autoreactive lymphocytes across the blood-brain barrier and the reduced function of regulatory T cells trigger autoimmunity in MS [2,3].

During the activation of T cells, interleukin 2 (IL-2) initiates the progression of the cell cycle via the E2F transcription factor family. This family consists of activator class I (E2F1-3), repressor class II (E2F4-5), and two further E2Fs (6 and 7), whose function is less well known. It is a member of the transcription factor E2F1 and forms heterodimers with another transcription factor, DP1. E2F1 is normally an important regulator of proliferation, differentiation, and apoptosis of T cells in the cell cycle. The elimination of mature and immature reactive T cells by apoptosis plays an important role in the prevention of autoimmunity [4].

Experimental studies have shown that T-cell responses are impaired in mice with reduced E2F1 gene expression. It is assumed that the E2F signaling pathway plays a role in autoimmunity by influencing the progression of cells in the cell cycle. It was found that autoimmunity does not occur in mice with E2F1 deficiency [5].

E2F1 has been shown to mediate the proliferation of immune cells triggered by HTLV-1, the virus that causes HTLV-1-associated myelopathy, an MS-like disease [6].

This study aims to evaluate E2F1 protein expression levels in blood samples from MS patients and to compare these levels with the clinical characteristics of the patients and those of a control group. E2F1 levels were investigated in experimental autoimmune encephalitis models [6]. To our knowledge, however, there are no studies in the literature that examine the protein concentrations of these genes in MS patients.

## Materials and methods

Methods

Participants

Patients over the age of 18 who were diagnosed with MS according to the McDonald criteria revised in 2017 [7], who were followed up in the neurology outpatient clinic between January 2023 and January 2024, and who agreed to participate in the study were included. Patients with a chronic disease other than MS and patients with a systemic inflammatory disease or another autoimmune disease were excluded. The medical information and examination results of the patients admitted to the outpatient clinics of SANKO University School of Medicine, Department of Neurology and Dr. Ersin Arslan Training and Research Hospital were collected by scanning their medical records. A control group that met the criteria for age and gender was recruited and their data was recorded.

ELISA

10-15 ml of venous blood from MS patients and the control group were collected in EDTA tubes. The blood samples were centrifuged at 2500 g for 10 minutes and the sera obtained were stored at -20°C.

The ELISA test was performed in 96-well plates, allowing a high throughput of results. The bottom of each well was coated with the corresponding antibody.

Each patient’s serum and control’s serum were incubated in individual wells. After allowing the serum to sit at room temperature for one hour, it was removed, and the wells were thoroughly rinsed with buffers. Next, peroxidase enzymes were added for 30 minutes to facilitate the transformation of colorless substrates into colored products. In the final stage, an enzyme substrate was applied, producing a visible color change in the wells. Once the reaction concluded, the plate was read using an ELISA reader, measuring the optical density of each well at a wavelength of 450 nm.

The researchers who analyzed the serum levels completed the study and reported the results blinded to the clinical information associated with the samples.

Ethics Committee

All procedures involving human participants in this study adhered to the ethical standards set by both the institutional and national research committees and the 1964 Helsinki Declaration. Ethical approval was granted by the SANKO University Clinical Research Ethics Committee prior to the study's commencement (approval date: 19.01.2023, approval number: 2023/02-01).

Statistical Analysis

Descriptive statistics include the mean, standard deviation, median, and quartiles for continuous variables measured, while frequencies and percentages are provided for qualitative variables. The agreement of the continuous variables with the normal distribution was assessed using the Kolmogorov-Smirnov test. As no parametric test conditions were given for comparisons between two independent groups in relation to continuous variables specified by measurement, the Mann-Whitney U test was used, and the Kruskal-Wallis test was used for comparisons between more than two independent groups. The chi-square test was used for group comparisons of qualitative variables. The relationship between two continuous variables was assessed using the Spearman Rank Correlation Coefficient. p<0.05 was considered statistically significant. IBM SPSS Statistics for Windows, Version 23 (Released 2015; IBM Corp., Armonk, New York, United States) was used for data analysis.

## Results

Participants' characteristics

Twenty-four MS patients (17 women, seven men) aged 20-54 years (mean±SD=34.75±10.16) and 21 controls (13 women, eight men) aged 22-51 years (mean±SD=34.29±9.33) were included in the study. Of the patients, 17 had relapsing-remitting multiple sclerosis (RRMS), five had secondary progressive multiple sclerosis (SPMS) and two had primary progressive multiple sclerosis (PPMS). The demographic characteristics of the people with MS and the control groups are shown in Table 1.

**Table 1:** Demographic characteristics of the people with MS and control groups n: number; SD: standard deviation; BMI: body mass index; PwMS: people with multiple sclerosis

	PwMS (n=24)	Control (n= 21)	p
Age (mean±SS)	34.75±10.16	34.29±9.33	0.875
BMI (mean±SS)	25.96±5.75	24.26±3.79	0.255
Gender n (%)			
Male	7 (29.17)	8 (38.09)	0.697
Female	17 (70.83)	13 (61.91)	

In the patient group, the number of patients who had been diagnosed for five years or less was 10, and the number of patients who had been diagnosed for more than five years was 14. While 10 patients with MS had experienced relapses in the last two years, 14 had no history of relapses. The clinical and imaging findings in the MS patient group are listed in Table 2. In the comparison of E2F1 protein levels between patient and control groups by gender, no significant difference was found among men; however, women in the MS group showed statistically higher levels (p=0.232, p=0.028).

**Table 2:** Clinical and imaging findings in the MS patient group n: number; SD: standard deviation; min-max: minimum-maximum; MS: multiple sclerosis; DMT: disease-modifying treatment; MRI: magnetic resonance imaging; EDSS: Expanded Disability Status Scale.

People with MS (n=24)	n (%)
MS type	
Relapsing-remitting MS	17 (70.8)
Secondary progressive MS	5 (20.8)
Primary progressive MS	2 (8.3)
Clinical at the first attack	
Optic neuritis	3 (12.5)
Motor	8 (33.3)
Sensory	3 (12.5)
Motor+ sensory	4 (16.7)
Brainstem/Cerebellar	6 (25.0)
Neurological examination	
Normal	11 (45.8)
Abnormal	13 (54.2)
DMT	
Yes	22 (91.7)
No	2 (8.3)
Localization of lesions in MRI of MS patients	
Cortical/Juxtacortical	19 (79.2)
Periventricular	22 (91.7)
Infratentorial	10 (41.7)
Spinal cord	18 (75)
EDSS	
Mean ± SD	2.22 ± 2.54
Median (min-max)	1.5 (0.0 - 9.5)

**Figure 1:**
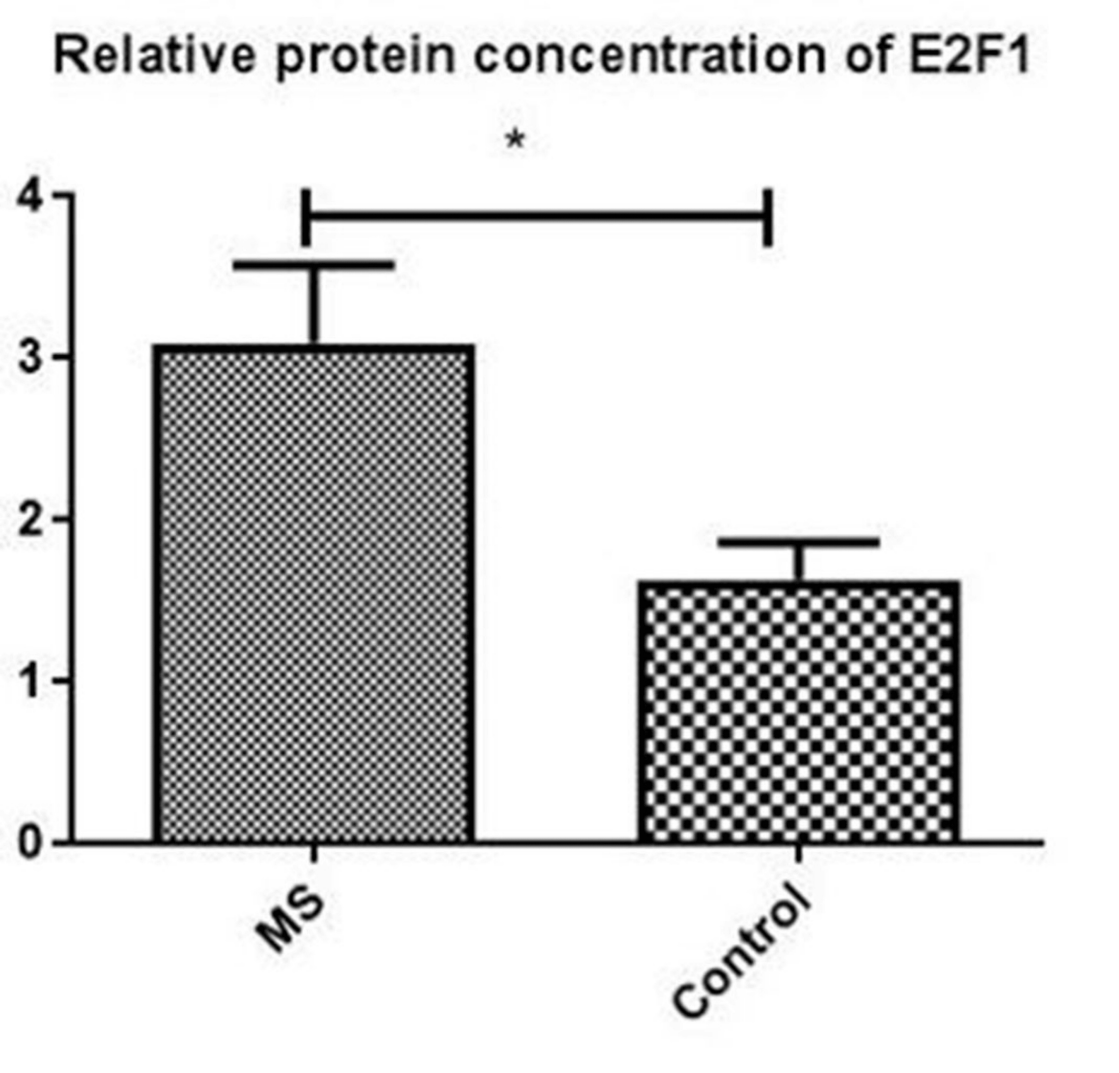
E2F1 levels in people with multiple sclerosis and controls MS: multiple sclerosis * indicates p=0.011

E2F1 protein levels were higher in the MS group than in the control group (p=0.011) (Figure 1). E2F1 protein levels were found to be significantly higher in RRMS (n=17) than in progressive MS [SPMS (n=5) + PPMS (n=2)] (p=0.024) (Table 3).

**Table 3:** Comparison of serum E2F1 levels by the MS type n: number; Min-max: minimum-maximum; MS: multiple sclerosis.

		Relapsing-remitting MS (n=17)	Primary and secondary progressive MS (n=7)	p
E2F1 (n=24)	Min-max	1.07-9.61	1.05-2.12	0.024
	Median [25%-75%]	2.80 (1.95-4.12)	1.77 (1.15-2.07)	

No association was found between the E2F1 protein level and time to diagnosis, EDSS, age at diagnosis, time to first relapse and duration of treatment (p>0.05).

## Discussion

This study showed that the E2F1 protein levels in MS patients were significantly higher than in the control group. In addition, E2F1 protein levels were found to be higher in the RRMS subgroup than in the progressive MS subgroups (SPMS and PPMS). These results suggest that the transcription factor E2F1 may play a potential role in the pathophysiology of MS.

It has been suggested that E2F1 may have effects on autoimmunity at the molecular level in vivo. The role of E2F1 in autoimmune diseases has been associated with cell cycle regulation and apoptosis [8-10]. Studies have shown that there are irregularities in the E2F signaling pathway in patients with MS [11,12].

It has been shown in the literature that E2F1 is an important regulator of apoptosis of immune cells and plays a special role in the control of autoimmune reactions [8,13]. It has also been shown that the activated E2F pathway can promote the cell cycle toward a Th1 cell phenotype and thus enhance autoimmunity. Experimental studies have shown that E2F1 -/- mice exhibit apoptosis of immune cells. The E2F1 signaling pathway is thought to have a positive effect on the lifespan of immune cells. E2F1 genes encode proteins such as cyclin E, dihydrofolate reductase (DHFR), and E2F1, which act at the transition from the G1 to the S phase of the cell cycle [8]. It has been suggested that defects in cell cycle progression and apoptosis of immune cells in the absence of E2F1 or E2F2 together with E2F1 play a role in the development of lupus-like disease [8,14,15].

In the autoimmune disease rheumatoid arthritis (RA), overexpression of E2F1 has also been shown to suppress the proliferation and invasion of RA fibroblast-like synoviocytes and the production of proinflammatory cytokines via the p53 signaling pathway, and it has been suggested that this may offer new targets for treatment [16]. In this context, the hypothesis that dysregulation of E2F1 in MS may trigger the inflammatory responses of the disease is consistent with the literature. However, the effects of E2F1 on the pathogenesis of MS need to be further investigated.

The results of our study suggest that E2F1 could serve as a potential biomarker in MS patients. Elevated levels of E2F1, particularly in RRMS patients, appear to be associated with disease relapses, suggesting a role in active inflammation. Further studies are needed to confirm the potential of E2F1 as a tool for monitoring response to treatment or predicting disease progression. In addition, therapies targeting the E2F1 signaling pathway may offer promising new approaches for the treatment of autoimmune diseases such as MS.

This study was performed on a small sample and the results need to be confirmed and studied in larger populations. In addition, the ELISA method used may have limited ability to detect all variants of the protein. Limitations of our study include that serum biomarkers, including E2F1, may not directly reflect changes in the CNS and may be affected by factors such as diurnal variation. In our study, clinical parameters such as age and disease duration showed no significant association with E2F1 levels, but these results should be re-evaluated in larger studies. In particular, further investigation of E2F1 levels in different clinical subgroups of MS will help us to better understand the role of this protein in the pathophysiology of the disease.

## Conclusions

This study is one of the first to investigate the role of the E2F1 protein in MS and provides valuable insights in this field. Studying E2F1 and related proteins in larger patient populations will lead to a deeper understanding of the immunological processes that trigger MS. In addition, investigating the role of E2F1 in different MS subtypes is crucial to clarify its involvement in the clinical variability of the disease. Finally, therapies targeting E2F1 could open new avenues for the treatment of MS and related autoimmune diseases.
